# ASReview LAB v.2: Open-source text screening with multiple agents and a crowd of experts

**DOI:** 10.1016/j.patter.2025.101318

**Published:** 2025-07-03

**Authors:** Jonathan de Bruin, Peter Lombaers, Casper Kaandorp, Jelle Teijema, Timo van der Kuil, Berke Yazan, Angie Dong, Rens van de Schoot

**Affiliations:** 1 Department of Research and Data Management Services, Information Technology Services, Utrecht University, Utrecht, the Netherlands; 2 IDfuse, Utrecht 3526 KS, the Netherlands; 3 Department of Methodology and Statistics, Faculty of Social and Behavioral Sciences, Utrecht University, Utrecht, the Netherlands

**Keywords:** systematic reviews, machine learning, multiagent systems, crowdsourcing, transparency, reproducibility, hyperparameter optimization, active learning, data-driven screening, open-source software

## Abstract

ASReview LAB v.2 introduces an advancement in AI-assisted systematic reviewing by enabling collaborative screening with multiple experts (“a crowd of oracles”) using a shared AI model. The platform supports multiple AI agents within the same project, allowing users to switch between fast general-purpose models and domain-specific, semantic, or multilingual transformer models. Leveraging the SYNERGY benchmark dataset, performance has improved significantly, showing a 24.1% reduction in loss compared to version 1 through model improvements and hyperparameter tuning. ASReview LAB v.2 follows user-centric design principles and offers reproducible, transparent workflows. It logs key configuration and annotation data while balancing full model traceability with efficient storage. Future developments include automated model switching based on performance metrics, noise-robust learning, and ensemble-based decision-making.

## Highlights

• ASReview LAB v.2 supports multiagent AI-assisted systematic reviewing with expert crowds
• Users can switch between fast, domain-specific, and multilingual models
• Model improvements using the SYNERGY benchmark led to a 24.1% reduction in loss
• Transparent, reproducible workflows balance model traceability with practical storage

## Teaser

ASReview LAB v.2 enables efficient, transparent AI-assisted systematic reviews by combining multiple machine learning models with input from expert crowds. Users can switch between fast, general-purpose, and domain-specific models within the same project. Leveraging the SYNERGY benchmark, version 2 improves performance by 24.1%. The open-source platform paves the way for scalable, collaborative knowledge curation and demonstrates how AI can support expert judgment in high-stakes decision-making across science, medicine, and policy.

## Introduction

Accurate and trustworthy information is vital for knowledge sharing and decision-making. This need is even greater now that the volume of available text is growing faster than ever. In parallel, deliberate campaigns spread misinformation, disinformation, and mal-information, which can quickly circulate on social media. These trends raise questions about the credibility of journalists, researchers, government bodies, and other key knowledge agents. The combination of the influx of text data with the increase in noisy data has made the task of systematic screening a resource-heavy task. Although recent advances in large language models (LLMs) have improved certain text-processing capabilities, they cannot fully replace human oversight. At the same time, human experts alone cannot keep pace with the rapid influx of data. These challenges call for a new generation of machine learning tools that combine human expertise with AI-driven efficiency.

Several strategies exist to cope with the ever-growing influx of research articles. One approach is reducing dataset size using narrower filter criteria.^1^ However, this can lead to missing key studies.^2^ Another tactic is to delegate screening entirely to LLMs.^3^^,^^4^^,^^5^ Yet, LLMs are prone to “hallucinations” and can produce false outputs.^6^^,^^7^ We, therefore, strongly argue that humans must remain the oracles, the final decision-makers, in modern research applications, with LLMs serving as a potential quality check^8^ or for passage retrieval and document retrieval,^9^ among many other useful applications.

Active learning^10^ represents a more robust solution. The information retrieval (IR) task is to find all relevant records by querying specific records proposed by a learner. In a classical active learning setup, an AI agent makes screening recommendations, and a human-in-the-loop (the “oracle”) validates them to optimize the performance of the model. In the recent literature, active learning to rank (ALTR)^11^ has been proposed, in which an annotator, a human expert, interactively queries the active learner (also called AI agent or ranking agent) and ranks the unlabeled data. Researcher-in-the-loop active learning^12^ is a sub-case of ALTR, where the oracle (human screener) iteratively requests the highest-ranked records with the goal of retrieving the relevant ones as quickly as possible. Many simulation studies show that this approach outperforms random sampling and manual screening of all documents^13^; see Teijema et al. for a systematic review.^14^

As we strongly believe in open science, our vision led to the birth of ASReview project—which led to ASReview LAB, the open-source software for efficient and transparent systematic reviews written in Python. In 2017, an initial research grant enabled the development of a prototype, culminating in the first GitHub release (v.0.1.0) in 2019. Building on feedback from early adopters, version 0.9 introduced a user-friendly interface, and by 2020, the v.0.11 release marked a major step forward in usability. In 2021, the framework was described by Van de Schoot et al.,^12^ which significantly boosted interest and funding, enabling the stable version 1 series, first released in June 2022. Subsequently, server deployment capabilities were extended (https://github.com/asreview/asreview-server-stack) for easy and secure server deployment. Throughout this development, a community of users has actively contributed features, discussed improvements, and shaped the software’s ongoing development (https://github.com/asreview/asreview/discussions). For a comparison of features between the major versions, see Table 1.

**Table 1:** A comparison of features between the major ASReview versions

Feature	v.0.9+	v.1.x	v.2.0
**Front-end features**			

UI based on material design	V	V	V
Browser UI	V	V	V
Dashboard	X	V	V
Dark mode	X	V	V
Mobile device optimization	X	V	V
Model transparency components	X	X	V
Labeling history visualizations	X	X	V
Tooltips for each component	X	X	V
Stopping suggestions	X	X	V
Accessibility optimization	X	X	V
Quick setup	X	X	V
Add tags	X	X	V
Dashboard	X	X	V

**Technical features**			

Active learners	V	V	V
Crowd screening	X	X	V
Model switching within a project	X	X	V
Quick setup	X	X	V
Start with random screening	X	X	V

**Platform features**			

Server installable	V	V	V
Account creation	X	V	V
OAuth authentication	X	V	V
SAML	X	X	V
Team creation	X	X	V

V, present; X, not present; SAML, security assertion markup language; UI, user interface.

Numerous other machine learning-assisted tools have emerged to aid systematic reviews, including Abstrackr,^15^ Colandr,^16^ Rayyan,^17^ RobotAnalyst,^18^ Research Screener,^19^ DistillerSR,^20^ and RobotReviewer.^21^ While these solutions have achieved success, many remain closed-source algorithms with limited or no interoperability. Moreover, software operating on server data might be user friendly unless it is not transparent about what is done with the data stored and processed by the servers. In an era of open science, this lack of transparency and user data ownership has become a significant drawback,^22^^,^^23^ and tools like ASReview, but also DenseReviewer^24^ and FASTREAD,^25^ show that it is possible to develop open-source software. Furthermore, as concluded in a systematic review^14^ and demonstrated in an extensive simulation study comparing 100+ models,^26^ different data require different models for optimal retrieval of the relevant documents, and only ASReview has integrated a wide range of models and is flexible enough for users to implement their own model.

So, ASReview has demonstrated its ability to speed up systematic reviews.^26^^,^^27^^,^^28^^,^^29^^,^^30^^,^^31^^,^^32^^,^^33^^,^^34^^,^^35^^,^^36^ Yet, with the continuous growth of textual data, the software must further evolve to meet user demands. Version 2 introduces a multiagent system^37^^,^^38^ in which AI agents propose records to the expert serving as an oracle making the final labeling decision. Each agent specializes in different tasks, such as other features of the texts (e.g., short versus long abstracts or domain-specific language) or different stopping heuristics.^39^ This setup reduces the risk of missing entire classes of relevant papers. Moreover, advanced transformer-based models are gaining popularity but generally require large (labeled) datasets. These can be more quickly generated by a group of expert screeners rather than a single individual. The multiexpert approach blends the power of crowd screening^40^^,^^41^ with the Screenathon approach,^42^ where experts work together in large consortia. Therefore, ASReview v.2 offers the possibility of using a crowd of experts to label large amounts of textual data while jointly training AI models.

The current paper introduces the underlying infrastructure for this multiagent, multiexpert crowd solution offered in ASReview v.2.0. We also present the simulation framework to identify the agent best suited to a particular dataset, ensuring that systematic reviews are more efficient and comprehensive. We present the results of a simulation study optimizing the new default model of v.2. In what follows, we first describe data requirements, followed by how we propose to map the AI-agent terminology onto the ALTR system as is used in ASReview for screening prioritization. We provide a detailed implementation of the software, followed by statistical examples, back-end code, and front-end screenshots. We first present this information for the screening project, followed by a simulation with ASReview. We provide an overview of the different layers of the software and how extensions fit into the framework. Finally, we present some future developments that pave the way for the next version.

## The multiagent, multiexpert system

### Background

ASReview’s active learning framework, as introduced in van de Schoot et al.,^12^ maps naturally to the literature on AI agents.^37^^,^^38^ In its most essential environment is a pool of unlabeled records and user responses indicating which records are relevant. The system’s goal, or reward, is to identify a collection of potentially relevant records as efficiently as possible (maximizing recall). The system acts as a “manager of agents,” the so-called active learner, deciding which agent’s policy to use at each stage of the learning cycle. At any moment, one agent is active, and that agent’s decision-making policy determines the ranking of the unlabeled records, which can be requested by an expert, and the hand-off or stopping procedures.

The agents’ model for one cycle of the active learner consists of a combination of a feature extractor transforming text data into vectors, a classifier predicting relevance scores, a balancer dealing with the spare number of relevant records, and a querier to determine which records to label next (for example, certainty, uncertainty sampling, or a mix of both). The combination of the four components is tuned for optimal performance (i.e., hyperparameter tuning). A ranking algorithm puts all unseen records in the queue, and the agent takes action by rank-ordering all records from the pool to present them to the expert (i.e., the human reviewer). After receiving new labels from the expert, it re-trains its classifier and updates the rank order—much like an AI agent refining its policy after seeing the consequences of its previous decisions.

While ASReview version 1 shows the highest-ranked record to just one oracle, in ASReview version 2, the highest-ranked records are distributed across the screening crowd (if available). The experts in the screening crowd act as *oracles*, each providing labels to the highest-ranked record from the pool available to them at that point in time. Each newly labeled record informs the agent about what is relevant, guiding subsequent rankings of the pool. Specifically, every time a new label arrives from the crowd, a task server checks if a worker for the project is free and potentially triggers another iteration of the learning cycle. If all workers in the project are occupied, the task server will wait for the next label to arrive. This way, labeling and re-training models occur asynchronously, resulting in a *dynamic re-ranking* of the pool without any lag for the users.

Each agent runs until a switch condition is met, then hands over the environment to another AI agent. For example, one agent begins with an initial model combination (agent A) that’s fast and has shown good performance with a limited training set (e.g., an SVM classifier on TF-IDF features). The agent actively queries the crowd of experts for labels, and as the crowd generates more records, the labeled dataset grows. After a certain point—e.g., after collecting *k* labels—it *hands off* control to another agent. Agent B is initialized (e.g., using and training a new classifier on features from MXBAI) using all the labeled data accumulated so far, as the new agent can ingest the labeled dataset from the previous cycle. From now on, agent B will decide which records to present for labeling, and if agent B depletes, you can hand off to other AI agents.

The system concludes the active learner cycles once a stopping condition (or termination condition) is reached.^32^^,^^39^^,^^43^ There is also the global stopping condition next to the cycle’s stopping condition, for example, when the end of data is reached or if no more agents are available. Finally, a user can terminate the system and mark the project as finished. After the global stopping condition is met, all labeled records are consolidated, and the collection of relevant records is exported for subsequent steps in the systematic review.

### Implementation

**Figure 1:**
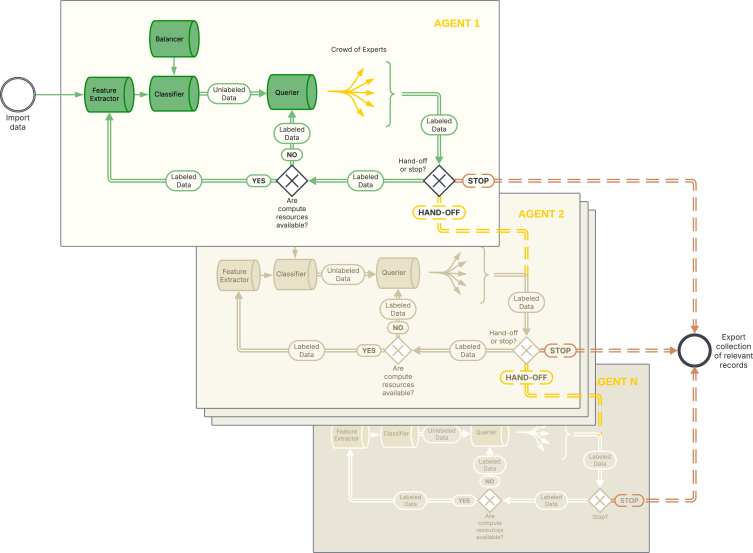
Schematic overview of the multiagent, multiexpert screening cycle in ASReview v.2

Below is a concise, step-by-step overview of one active learning cycle; see also Figure 1.

(1) Load data and initialize the project
  ○ Load a dataset containing titles and text to be screened. A project will be created, including metadata like the name of the project, the date of creation, and the user name.
  ○ Optionally, adjust the default model.
  ○ Optionally, select a set of pre-labeled records (“priors”) for initial training data—if none are provided, by default, the first agent presents random records until a minimum training set size is reached with one relevant and one irrelevant record, after which the default model is initiated.
(2) Check hand-off or stop condition
  ○ Each agent starts by determining whether a global stopping condition has been met (e.g., all records are labeled) or if a stopping condition is satisfied that will trigger the hand-off to the next agent.
  ○ If the stop condition is triggered, proceed accordingly (i.e., terminate the system or hand off to the next agent). If not, proceed to the next step.
(3) Transform data
  ○ Check if the features needed for the current agent are available. Previous features will remain available for future re-use.
  ○ If not, transform text using the agents’ feature extractor (e.g., TF-IDF or MXBAI). A cached version is used if available.
(4) Train the classifier
  ○ Compute or estimate sample weights given by the agents’ balancer.
  ○ Train the agents’ classifier on labeled records to produce probability (for, e.g., logistic regression) or decision scores (for, e.g., SVM) for unlabeled records.
(5) Rank records
  ○ Use the agents’ querying algorithm to rank the pool (the unlabeled records), for example:
    ⁃ Certainty sampling: rank from highest to lowest probability of relevance.
    ⁃ Uncertainty sampling: rank records to the decision boundary (e.g., probability ≈0.5 for logistic).
    ⁃ Hybrid: a combination of (un)certainty with mixing 5% random records.
    ⁃ Top-down selection as is available in the initial dataset (if selected, no model training will be triggered).
    ⁃ Random selection (this is the default option if the minimum training set is unavailable, and once there is enough training data, the selected ranking based on the classifier will be used; if selected by the user, no model training is done).
(6) Expert query records
  ○ The ranked pool of records serves as a queue.
  ○ Each human annotator that requests a record to label receives the highest-ranked record not requested by one of the other annotators.
(7) Annotate records
  ○ Experts label their records as an oracle: “relevant” or “not relevant” (and might add tags or notes).
  ○ Relevant records are added to the relevant collection.
  ○ The LAB server requests the next record from the ranked pool.
(8) Model training on task server
  ○ The task server is notified whenever new records are labeled.
  ○ If compute resources (workers) are available (i.e., cores on your machine with the default set to 2), the classifier is re-trained and it updates the ranking of unlabeled records.
(9) Hand-off or stop
  ○ If the stop condition (e.g., a certain number of labeled records) is met, hand over control to the next agent, returning to step 2.
  ○ If there is no next agent, or if the global stopping criterion is reached (e.g., all records labeled), export the complete set of labeled data—particularly the relevant collection—and end the process.

Consider an example: let K be the entire dataset, where $K_{u}$ is the set of unlabeled records, and let k be a batch of records presented to the crowd of experts, where $k^{+}$ and $k^{-}$ are the newly labeled relevant and irrelevant records provided by the crowd. Initially, you may have some prior labels in K (i.e., $K^{+}$ or $K^{-}$) or none at all, so the system begins with $K=K_{u}\cup K^{+}\cup K^{-}$. For a screening project, it is assumed that the system starts with at least ${K=K}_{u}$, whereas for a simulation project (next section), a fully labeled dataset is assumed, $K=K^{+}\wedge K^{-}$. When the system is in operation, the labels of the records for $k^{+}$ and $k^{-}$ are added to.

Furthermore, let $A=A_{1},A_{2},{\ldots ,A}_{n}$ be a set of agents, the active learner, with $A_{n}$ being the last agent in the set. Each agent $A_{i}$, taking actions within one cycle, has a set of stopping rules for handing off to the next agent and a global stopping policy. The hand-off policy can be different for each $A_{i}$ and will trigger the next agent in the system. When the global stopping condition is triggered, for any $A_{i}$, but always for $A_{n}$, the active learner system terminates, and that agent exports the collected set $K^{+}$. The user can also trigger SystemStop(t) at any time t and mark the project as “finished.”

Suppose you have no initial labels, so ${K=K}_{u}$ and the task is to stop when all relevant records in K are found, and you want to stop before k=K (to save screening time). Furthermore, suppose there are n=4 agents and that these operate sequentially. Every agent starts by checking whether the stopping rule for its hand-off policy and the global stopping rule have been met. As the global stopping rule, we use k=K and $A_{n+1}=\emptyset$. Hand-off control to the next agent is based on specific policies, for example:

(1) For the first agent, hand off if k=100 (i.e., the crowd labeling 100 random records), following the calibration step of the “SAFE” procedure^39^ (see, for a crowd-based application of the Screenathon procedure, Monschau et al.^42^). The first agent uses no model; instead, it ranks records via a random query strategy and puts the records in the queue. Each free annotator requests the highest-ranked record, until

$$\text{if}\;k=100\;\text{then}\left\{ \begin{array}{c} \text{if}\;k=K\cup A_{n+1}=\emptyset ,\text{STOP}\;\text{and}\;\text{EXPORT}\;K^{+},\\ \text{else},\text{STOP}\;\text{and}\;\text{HAND}-\text{OFF}\;\text{to}\;A_{n+1}. \end{array}\right.$$

(2) The second agent starts with $K^{\prime }=K_{u}\cap {\left[K^{+}\cup K^{-}\right]}_{\left(100\right)}$ and hands off if $K^{\prime }$ is at least 5% of K (and checks for the global stopping rule).
(3) For the third agent, we select the default model, and the agent first checks if a feature matrix is available; if not, it applies a feature extractor (e.g., TF-IDF). Then, it checks if a classifier is able to be fitted (and checks for the global stopping rule), so $K^{\prime \prime }=K_{u}\cup {\left[K^{+}\cap K^{-}\right]}_{\left(100+5\%\right)}$ with at least a minimum training set size of $\left|K^{+}\right|\geq 1\cap \left|K^{-}\right|\geq 1$. If there are insufficient data, it continues gathering random labels, and once there are, it triggers the classifier. While the classifier is training, the crowd can continue screening and adding labels. Whenever the task server has new results, it will update the pool according to the new ranking, this time based on the model results. For the hand-off condition, let Δk be a batch of consecutive labeled records with the same label, in our case, $k^{-}$. The hand-off policy is Δk≥100; so, the policy of this agent is to present unlabeled records to the crowd until 100 consecutive irrelevant records are labeled, then it hands off (and checks for the global stopping rule).
(4) The last agent follows the model switching procedure,^30^ applying an MXBAI^44^ model as the vectorization method and SVM as the classifier. While training the new features, the crowd can continue adding labels based on the ranking from the previous model so as not to waste any time. The ranking will be updated as soon as the new model results are available. The policy of this agent is to present unlabeled records to the crowd until the crowd labels 100 irrelevant records in a row. Since $A_{n+1}=\emptyset$, the agent stops the system after the stopping criterion is met and exports the collection of relevant records. So, $\text{if}\;\left|\Delta k\right|\geq 100\;⟹\text{STOP}\;\text{and}\;\text{EXPORT}\;K^{+}$.

### Default models and their hyperparameters

Users can choose from pre-set model combinations for which we optimized performance, customize their own set of model components, or add their own model.

The first pre-set model combination is the ELAS-Ultra series, where ELAS refers to the Electronic Learning Assistant, ASReview’s mascot and story-telling figure (https://github.com/asreview/asreview-artwork). Ultra refers to a series of established, fast, and excellently performing model combinations. Its hyperparameters are optimized on 24 systematic review datasets in the SYNERGY dataset.^45^ From each of the selected datasets, 10 prior combinations were sampled as a test set using a geometric distribution (*p* = 0.5) to address prior selection bias. In addition to this test set, a validation set was sampled in the same way to test generalization performance and prevent overfitting during hyperparameter tuning. Optimization was conducted leveraging the Optuna package^46^ (https://github.com/asreview/asreview-optuna), with the goal of minimizing the mean loss across all datasets and prior combinations; see Van der Kuil et al. for the data, scripts, and results.^47^

To reduce the search space, an exploratory parameter search was initially done for each combination of feature extractors (OneHot and TF-IDF) and classifiers (random forest, SVM, naive Bayes, and logistic regression). SVM appeared to use significant computational time; therefore, we switched from the scikit-learn support vector classifier to the linear support vector classifier.^48^ Moreover, we improved the balancing strategy in ASReview v.2, which decides how much to boost or penalize data points during training. Last, the TF-IDF feature representation between v.1 and v.2 was changed by adjusting the ngram_range hyperparameter: switching from unigrams (single words) in v.1 to uni- and bigrams (single words and pairs of consecutive words) in v.2. Based on the performance during this exploratory search, we focused our further optimization on the two top-performing combinations: TF-IDF with naive Bayes and TF-IDF with SVM. Table 2 presents a comparison of the mean validation loss (±SD) across all 24 datasets and 10 prior combinations. The results show that the mean loss for each v.2 model is lower than that of any v.1 model. If we look at the two default models (marked by superscript a) we see a 24.1% decrease in mean loss between the default models of v.1 and v.2. Moreover, the standard deviation is consistently lower for v.2 models, indicating more consistent performance across datasets. As a result, we have chosen the improved implementation of SVM plus TF-IDF as the new default: ELAS-Ultra.

**Table 2:** Mean loss values for the old and new default models with, in parentheses, the standard deviation across 10 prior combinations

	ASReview v.1, M (SD)	ASReview v.2, M (SD)
TF-IDF + naive Bayes	**0.0821 (0.0081)**^a^	0.0757 (0.0073)
TF-IDF + SVM^b^	0.0875 (0.0083)	**0.0623 (0.0040)**^a^
MXBAI (+SVM)	N/A^c^	0.0610 (0.0046)
E5 (+SVM)	N/A^c^	0.0640 (0.0043)

a Default model.

b Version 1 uses the SVC implementation, v.2 uses the LInearSVC implementation.

c The MXBA I and E5 models were not available in v.1.

**Figure 2:**
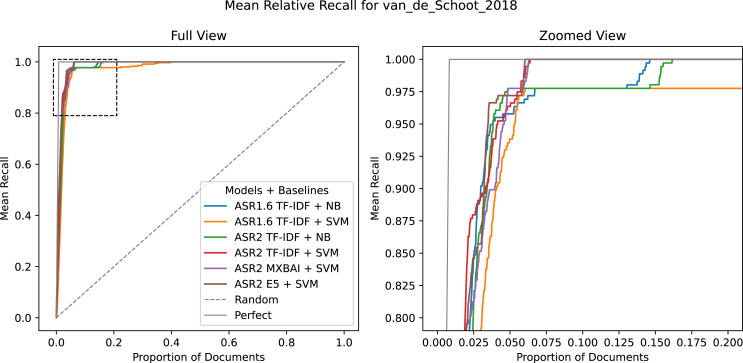
Recall plots comparing the default models in v.1 with those in v.2

To illustrate this improvement in practical terms, we compared the recall performance across the two model configurations and ASReview v.1 and v.2 on a systematic review of trajectories of post-traumatic stress disorder (PTSD) after traumatic events,^49^ a representative systematic review from the SYNERGY collection. The authors labeled 4,455 papers for the initial review to include 38 relevant ones in their systematic review. When using the default model of ASReview v.1 to simulate this review for the validation set (10 prior combinations for this dataset), all relevant ones would have been found after screening 542.1 papers out of 4,455 (±189.47). With the further optimized Ultra model in v.2, only 270.6 papers (±11.48) would need to be screened. Recall curves in Figure 2 show the cumulative recall of the relevant records found as a function of the number of screened documents, providing insight into the efficiency of each of the model configurations. Figure 2 shows the recall curves for the two configurations in ASReview 1 and 2. Both ASReview 2 configurations retrieve relevant studies earlier than their ASReview 1 counterparts.

The second pre-set model combination, available via the Dory extension, ELAS-heavy, employs MXBAI optimized for retrieving records based on the records’ semantics. The same hyperparameter optimization protocol described earlier based on the SYNERGY collection was used to optimize the parameters, resulting in a slightly lower loss (see Table 2). For the PTSD data, it would reduce the number of papers to screen to 266.8 (±25.84), but at the price of higher computation time to obtain the vectors: ±15 min on a MacBook Air M2, whereas TF-IDF would take seconds. Therefore, we foresee that this model can serve as a model to switch to if enough labels have been gathered,^30^ as a quality check,^39^ or if a previously labeled dataset is available, for example, in an update of a systematic review (e.g., van de Schoot et al.^2^).

Third, as shown in Boer et al.,^34^ relevant texts written in non-English languages might be less optimally retrieved. Therefore, the third pre-set model is ELAS-lang, a multilingual model optimized for handling multiple languages at once based on Wang et al.,^50^ available via the Dory extension. The loss is comparable to that of the other models, and for the PTSD dataset, 269.9 (±2.88) records would need to be screened. Therefore, we can recommend ELAS-lang to users with datasets containing more than one language.

We continuously improve hyperparameters and update newer releases whenever we can find better parameters, always offering our users the maximum performance boost. We have implemented a versioned naming scheme for our pre-sets to maintain reproducibility while delivering the latest models and parameters. For instance, ELAS-u4 represents the current Ultra-series configuration, and future updates may introduce ELAS-u5 while retaining support for u4. Additionally, to replicate the performance of ASReview v.1 in v.2, we provide ELAS-u3, which uses the same model combination as the default in v.1. This naming scheme extends to all pre-set types: ultra, heavy, and lang. The most recent pre-sets are published at https://asreview.readthedocs.io/en/stable/lab/models.html.

Many other model combinations can be chosen, but not all combinations have optimal hyperparameters tuned. Moreover, users can also add new models via the template extension for new models, which provides a straightforward path for incorporating custom or emerging technologies (e.g., additional Hugging Face models). This flexibility ensures that ASReview remains at the cutting edge of machine learning techniques for systematic reviews.

Last, while the pre-set model combinations use the max query strategy, ranking based on most likely relevant records, the custom option can be used to select queries that sample random records, as in our example in the previous section. Currently, v.2 supports a single, but parameterized, balancer named Balanced, which adjusts the weight of data points based on their class label (relevant or irrelevant) to address class imbalance during training. However, the software is designed with extensibility in mind, allowing users to easily implement additional balancing strategies as needed.

### Data storage

Although ASReview LAB v.2 can reduce the time needed for systematic screening, it creates new challenges for transparency and reproducibility. Multiple AI agents are sequentially trained on an evolving set of labeled records contributed by a crowd of experts. As the labeling and training of the model occur asynchronously by the task server, it can become difficult to pinpoint which agent was actively recommending a specific record when one of the experts labeled it. Directly storing all intermediate model states from every agent would be prohibitively large—especially with modern transformer methods—yet omitting model data entirely prevents verification or replication of the screening process.

To address these concerns (see also Lombaers et al.^23^), ASReview maintains a project file that captures essential setup information, including the learner’s hyperparameters of the AI agent, the feature matrix or matrices, and any initially labeled records. This ensures that the starting configuration can be fully reconstructed. Additionally, all expert actions—such as the order in which records were labeled by which user_id, timestamps, annotator decisions, tags, and any notes—are stored. Because the multiagent system relies on these labeled records at each stage of model training, logging exactly how and when each label was provided is critical for retrospective analysis.

For model reproducibility, recording only the type of agent (e.g., the classifier plus its features), the number (or specific record IDs) of labeled records used in each training cycle used to train that classifier typically suffices to re-create the model’s predictions. Re-calculating these predictions involves re-training the same type of agent on the same labeled data at that iteration and will generate an identical or near-identical ranked list of unlabeled records. Some users may opt to store the full set of trained agent models for strict, byte-for-byte replication or where computational resources are limited. In most cases, however, storing all information about each agent’s training cycle with detailed results of the last iteration strikes an optimal balance between disk efficiency and review transparency, ensuring the multiagent screening process remains both streamlined and reproducible.^23^

Consider the slice of the project export in Table 3. The owner creates a screening project and imports a dataset without labels ($K_{u}=100$) and starts the first cycle with agent 1. After labeling one relevant (row 1) and one irrelevant record (row 2), the owner invites three more screeners to create a crowd of experts and selects the lightweight model ELAS-u4 with four pre-set model components. Agent 2 trains a model based on the training set of agent 1 ($K^{+}=1$ and $K^{-}=1$). Each expert requests the highest ranked record, so user 04 requests the highest ranked record, which is at that moment the fourth record of the first iteration of the cycle (row 6).

**Table 3:** Slice of the project export

	Labeling data				Training data			User data				
	Row no.	Record identifier	Label	Labeling time	Training set size	Record from queue	Model	User_id	Note	Tags		
										Exclusion reason 1	Exclusion reason 2	Discuss with team
**Agent 1**: STOP if $\left|K^{+}\right|\geq 1\cap \left|K^{-}\right|\geq 1$	1	145	1	2025-1-23 09:32:08.46	–	–	random	01	–	–	–	–
	2	56	0	2025-1-23 09:32:40.13	–	–	random	01	–	–	–	–
**Hand-off + invite crowd**												
**Agent 2**: STOP if k=15	3	120	1	2025-1-23 09:34:26.48	2	first of iteration 1 (It. 1)	ELAS-u4	01	–	–	–	–
	4	442	0	2025-1-23 09:34:26.55	2	second of It. 1	ELAS-u4	02	–	1	1	–
	5	4432	1	2025-1-23 09:33:26.55	2	third of It. 1	ELAS-u4	03	–	1	–	1
	6	13	0	2025-1-23 09:34:26.55	2	fourth of It. 1	ELAS-u4	04	–	–	1	–
	7	247	1	2025-1-23 09:35:27.11	2	fifth of It. 1	ELAS-u4	03	this is a note	–	–	–
	8	491	1	2025-1-23 09:40:28.43	6	first of It. 2	ELAS-u4	01	–	–	–	1
	9	102	0	2025-1-23 09:40:28.59	6	second of It. 2	ELAS-u4	04	–	1	–	–
	10	243	1	2025-1-23 09:40:30.12	6	third of It. 2	ELAS-u4	02	–	–	1	–
	11	86	1	2025-1-23 09:37:27.11	7	first of It. 3	ELAS-u4	03	–	–	–	–
	12	345	1	2025-1-23 09:47:27.11	10	first of It. 4	ELAS-u4	01	–	–	–	–
	13	34	1	2025-1-23 09:47:27.11	10	second of It. 4	ELAS-u4	04	–	–	–	–
	14	98	–	–	–	first of It. 5	ELAS-u4	02	–	–	–	–
	15	67	–	–	–	first of It. 6	ELAS-u4	04	–	–	–	–
	16	401	0	2025-1-23 09:58:40.22	14	second of It. 6	ELAS-u4	03	–	1	–	–
	17	76	–	–	14	third of It. 6	ELAS-u4	03	–	–	–	–
	18	279	0	2025-1-23 09:59:59.36	14	fourth of It. 6	ELAS-u4	01	–	1	1	1
**Hand-off**												
**Agent 3**: STOP if |Δk|≥100	19	76	0	2025-1-27 14:00:47.83	14	third of It. 6	ELAS-u4	03	–	–	1	–
	20	366	0	2025-1-27 14:02:47.83	15	first of It. 7	ELAS-h3	01	–	1	–	–
	…	–	0	…	…	…	ELAS-h3	–	–	–	–	–
	99	665	–	2025-1-27 16:02:47.83	15	99^th^ of It. 7	ELAS-h3	01	–	–	–	–
	100	45	0	2025-1-27 16:04:47.83	15	100^th^ of It. 7	ELAS-h3	03	–	1	1	–
$⟹\text{STOP}\;\text{and}\;\text{EXPORT}\;K^{+}=9$**.**												

User 03 labels their record first, and with this action triggers the task server, which starts to re-train the classifier (row 5). Since the labeling and training cycles are independent, user 03 does not have to wait for the model to be done training but simply requests the highest-ranked record at that time, which is the fifth record of iteration 1 (row 7). User 01 labels the record after the new model is done training and requests the highest ranked record, which is the first in the queue of iteration 2 (row 8), which has been trained on six labels ($K^{+}=4$ and $K^{-}=2$).

The labeling and training continue asynchronously until agent 2’s stopping condition is triggered by user 01; see row 18 (k=15). Agent 3 trains a new heavy model, ELAS-h3, while user 03 contributes a new label (row 19), but this record is not taken into account. After training the model, both users 01 and 03 continued labeling, and users 02 and 04 stop working on the project. In our example, the task server is in this stage set to never re-train the heavy model. With 100 records in the dataset and 3 records allocated but without a label (rows 14, 15, and 99), there are 97 labeled records, with $K^{+}=9$.

## ASReview infrastructure

**Figure 3:**
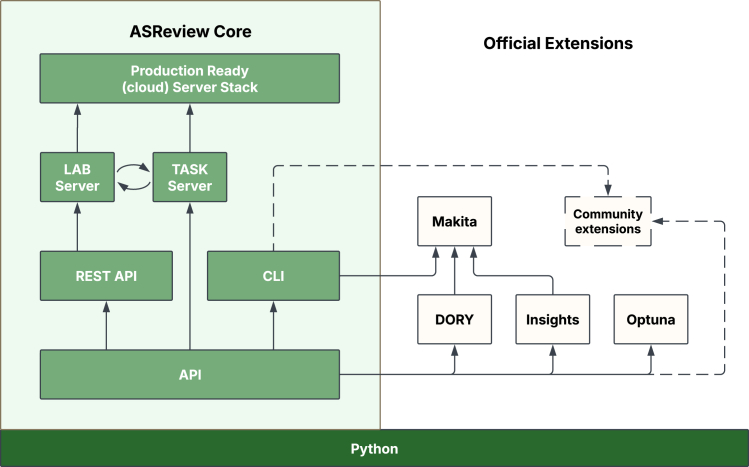
Overview of the ASReview infrastructure

Figure 3 shows an overview of the interoperable infrastructure of ASReview. The framework is largely written in Python and uses various other technologies such as Docker, WSGI, React, and SQL(ite). These components collectively form the infrastructure of the project, accompanied by official and community extensions that hook into the core via the application programming interface (API) or the command-line interface. Below, we describe the interfaces and servers used in the infrastructure, while the extensions are described later in our paper.

### Core interfaces

#### API

ASReview LAB ships with a documented API) that provides models, data, and project management functionality. The rich set of functions, classes, and modules allow researchers and developers to develop custom workflows, integrate new algorithms, or embed ASReview functionality in larger projects. It is also the foundation of the higher-level interfaces of ASReview LAB.

#### REST API

A stateless REST API written in Flask provides an interface for web applications built on ASReview. While integral to ASReview LAB, this REST API is still under active development and is not yet fully documented.

#### CLI

The command line interface (CLI) of ASReview provides an interface for users of computer terminals to start ASReview LAB, run simulations, list algorithms, and more. After installing ASReview via PyPI (https://pypi.org/project/asreview/), the command “asreview lab” will start the user-friendly web app interface written in React that is available in all major browsers.

Furthermore, “–help” lists available sub-commands, their origin package, and their version. It can also be extended with sub-commands provided by both official and community-built extensions. The general command structure is “asreview [-h] [-V] [subcommand].asreview.”

### Servers

#### Task server

ASReview LAB v.2 introduces a new task server for handling asynchronous tasks like training agents and running simulations. The task server comes with a network socket interface and makes use of Transmission Control Protocol (TCP) for communication. New tasks are sent to the task server, and the progress is logged. In its config file, you can set the port, the host, and the number of workers.

#### LAB server

The LAB server runs on Flask and serves the RESTful API.

#### Server stack

To streamline self-hosting and enterprise-level deployments, the ASReview server stack provides a production-ready Docker Compose setup https://asreview.readthedocs.io/en/stable/server/overview.html. This configuration packages the main components of ASReview—such as the AI engine (model server), the React front end, and a database layer—into separate, containerized services. As a result, organizations and users can run ASReview on their own hardware or in a cloud environment, ensuring data privacy and compliance with institutional policies. Although the ASReview team does not offer a managed hosting service, the server stack makes self-hosting straightforward. Users benefit from easier updates, robust scalability, and customizable authentication options, all while maintaining complete control over their infrastructure. This approach is particularly valuable for teams handling sensitive information or those who prefer to host their own software for security or regulatory reasons.

## Screening projects

### Input data requirements

ASReview supports datasets of textual records, such as titles and texts from scientific papers, news articles, or policy reports, acquired through a systematic search. Typically, only a small portion of these records will be relevant to our users, so the primary challenge is effectively identifying those records. The simulation infrastructure can work with any vectorized dataset, including image data.

Text data can be natively presented to ASReview in two main formats. First, tabular datasets are accepted in CSV, TSV, or XLSX formats. If records are already labeled, for example, from a previous study to serve as training data for the first iteration of the model, a column named “included” or “label” should indicate this, using 1 for relevant and 0 for irrelevant, while unlabeled entries remain blank. Second, RIS files exported from digital libraries (e.g., IEEE Xplore, or Scopus) or citation managers (e.g., Mendeley, RefWorks, Zotero, or EndNote) can also be used. If some records are already labeled, they should be stored in the “N1” field with tags “ASReview_relevant” and “ASReview_irrelevant” enabling re-import and continuation of ongoing screening using those labeled records as training data. More details on accepted fields, naming conventions, and partial re-import of labeled records are available in the documentation.^51^

Once the data are loaded, users can create a review project to screen unlabeled records or a simulation project to evaluate the performance of different models.

### Front end

**Figure 4:**
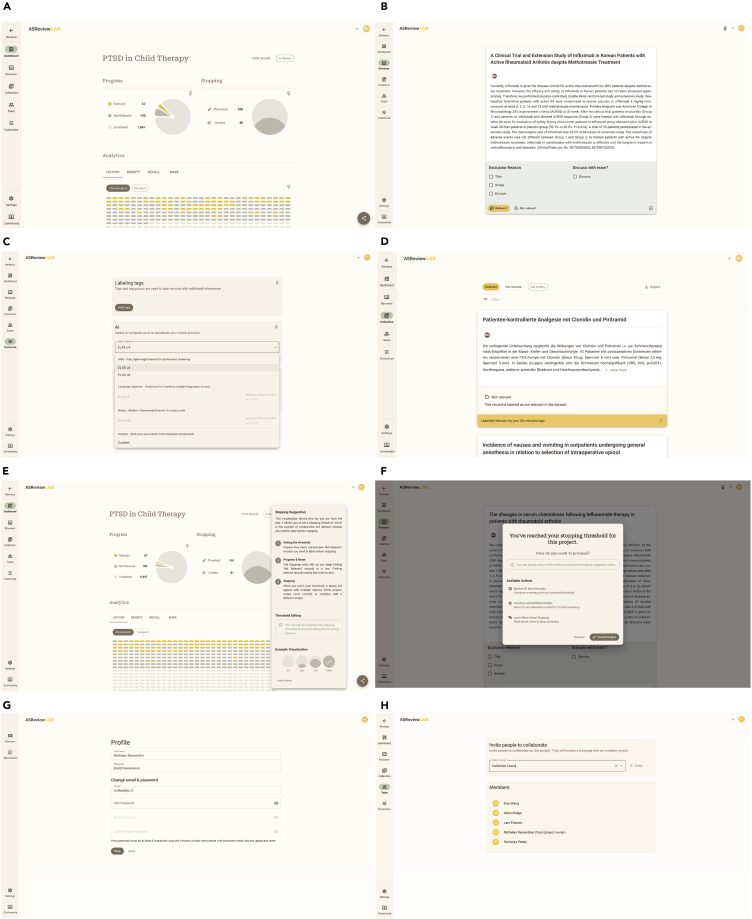
Screenshots of v.2 (A) How users can see different aspects of their progress in the project dashboard is illustrated. (B) The review screen with the labeling tags feature. (C) How users can select the agent learner in the project customization page is shown. (D) The collections page that includes previous labeling decisions. (E) How users can monitor the system’s stopping rules is shown—once a certain threshold is reached, the interface triggers a hand-off suggestion to another AI agent. (F) The hand-off suggestion is illustrated and the workflow for switching from one agent to the next is shown. (G) How users can view and edit their authenticated profile is illustrated. (H) The option to invite experts to the project is shown and the existing team members are displayed.

The ASReview LAB front end is written in React and implements the Material Design 3 design framework (https://m3.material.io/), ensuring a clean layout, intuitive navigation, and consistent interaction patterns; see some screenshots in Figure 4. Accessibility features are integrated by design; for example, color palettes have been chosen to accommodate users with varying degrees of color blindness, and a dark mode is supported for improved readability in low-light settings. With the implementation of the v.2 interface, it has been a deliberate choice to consult the *People + AI Guidebook* to emphasize the human-centered AI design aspects for ASReview LAB (https://pair.withgoogle.com/guidebook). By doing so, ASReview highlights the necessity of accommodating user needs as well as enhancing the explainability and trust with each iteration. Figure 4 contains some screenshots of the software, specifically the project dashboard, the review screen, learner agent selection, and the collections screen.

### Stopping suggestions

While ASReview version 1 relies entirely on the user’s judgment to decide when to stop screening, version 2 enables the user to define pre-determined stopping rules. Users can set their preferred stopping heuristic based on either a custom value of their choice or a percentage of their total records. This heuristic represents the amount of consecutive irrelevant records the user needs to label before receiving the hand-off suggestion dialogue. The stopping circle fills as the user keeps labeling irrelevant records in a row and re-sets to zero when a relevant record is found.

During the review, the dashboard provides real-time feedback about how close the user is to meeting the chosen stopping condition (see Figure 4). When the user reaches their threshold, a hand-off suggestion directs them to other possible AI agents. Current possible choices include reviewing 20 more records, continuing with a different agent learner, or finishing the project (see Figure 4).

### Invite experts

In the version 1 series, each project was tied to a single user. The ASReview server stack in version 1 allowed multiple users to create projects under one secure environment. An administrator could set up a private authentication database—through interactive tools or bulk uploading user details— allocating users to specific projects to which everyone must sign in before accessing their assigned projects.

By contrast, version 2 makes collaborative “crowd screening” possible, with a project owner who can invite multiple experts into a shared project. While owners have advanced privileges—for instance, they alone can delete a project—the assigned experts can jointly screen the records for that project. Each labeled record is screened only by one expert; hence, no interrater reliability can be computed. This is also not the goal of the crowd-screening option. Rather, the goal is to screen as many records as possible in a limited time, or it can be used for a Screenathon event,^42^ which is a literature-screening procedure that can be useful for large consortia crowdsourcing the screening to relevant stakeholders in a limited amount of time while forming a consensus about definitions relevant for the larger project and building a research community that lasts the project’s entire duration. It has been successfully used for the EU-funded IMPROVE project^52^ involving 26 partners across 10 European countries—spanning both academia and industry—to build an evidence-based framework for leveraging patient-generated health data in people-centered, integrated healthcare.

Figure 4 shows how a project can be initiated, and experts are invited to join the screening. After importing the dataset with potentially relevant documents, the crowd of experts can start screening. As soon as one of the screeners has met the stopping rule, all users will be notified after they label their record on the screen.

## Simulation projects

A simulation study can mimic the labeling process without depending on humans by leveraging an already labeled dataset, such as a previously published systematic review. Because the labels for all records are already known for a published paper, ASReview’s simulation software can automatically re-enact the screening process as if a human were labeling records in real-time interaction with the agent(s).

Simulating with ASReview LAB serves multiple purposes. First, if a user is unsure which model to select for a new (unlabeled) dataset, they can test how different learners perform on an existing labeled dataset that has similar characteristics. A user might also simulate a fully labeled dataset to see how much time could have been saved by using the system for future projects. Additionally, it can help detect “odd” relevant records^28^ that appear isolated from most other records but warrant closer inspection^29^ or to identify noisy labels.^35^ Moreover, it allows data science users to benchmark and validate new model components or entire sets of agents against those already implemented in ASReview, including combinations of stopping heuristics, switching between models, and combinations thereof. Alternatively, one could benchmark existing models but for different datasets.^26^^,^^30^^,^^31^^,^^32^^,^^33^^,^^34^

The simulation requires a fully labeled dataset K with both positive $K^{+}$ and negative $K^{-}$ labels: $K=K^{+}\cup K^{-}$. Users can choose to have ASReview LAB extract features directly from the imported dataset or provide a separate vector database. By default, the simulation study ends once all relevant records have been identified. At that point, metrics such as recall, loss, time to discovery,^27^ or work saved over sampling (WSS@95)^53^ can be calculated.

The simulation infrastructure is available via the API (for programmatic control), a CLI (providing advanced configuration), and the web app (with basic configuration options). This flexible design enables users of varying technical backgrounds to explore, refine, and benchmark how different models and parameters might perform before or after real-world screening. Additionally, simulation tasks can be parallelized and scaled on infrastructure like Kubernetes to handle extensive workloads.^31^ This approach maximizes computational resources and minimizes runtime, making it suitable for high-throughput or collaborative research projects.

### Simulate via the API

For more granular control over your simulation settings and workflows, you can directly access the ASReview Python API. This approach is especially useful if you want to implement custom models (e.g., experimental classifiers or feature extractors), to try out new sampling strategies beyond the defaults, or to integrate ASReview functionality into other data processing pipelines or larger software systems.

You can construct a simulation object through the API, configure learners and parameters, run the simulation programmatically, and capture detailed intermediate results. This level of flexibility empowers researchers and developers to tailor the active learning process to specific use cases or to rapidly prototype new methods. See the ASReview documentation^51^ and code examples on GitHub for detailed instructions on integrating your own AI components.

The simulation is initiated by

s = Simulate (df, y, learner),

where df is the data object supported by the feature extractor, y is the iterable containing labels, and learner is a list of agents with their feature extractor, the classifier with balancer, querier, and stopping criterion. Each learner component can have its own hyperparameters. Only the querier is mandatory for an agent; however, most agents will also have a feature extractor and classifier.

If needed, one can directly label certain records before starting, using

s.label([rec_i,rec_j,…]).

Then, the simulation is executed with the command

s.review().

The learner proceeds through multiple learning cycle iterations (as detailed in the previous section). Each cycle ranks the remaining records. Unlike human-based screening, the system automatically assigns true labels from K, with ${K^{+}=k}^{+}\cup {\;K}^{-}=k^{-}$, simulating what one annotator would do in a real-world workflow. After each label—or each batch of labels—the learner re-trains, refining the ranking.

The global stopping rule triggers once all relevant records in $K^{+}$ are identified (${K^{+}=k}^{+}$). Any remaining unlabeled records are then ranked based on the learner’s most recent iteration. The simulate object, s, retains an attribute named s._results, which captures the reproducible state of the active learning process.

### Simulate via the CLI

The command-line tool enables scalable simulations in ASReview. A basic simulation can be run via the terminal with

asreview simulate MY_DATASET.csv -s MY_SIMULATION.asreview.

Here, MY_DATASET.csv refers to a fully labeled dataset, and MY_SIMULATION.asreview is the output file that stores all simulation details and results. The default model will be used, and the simulation ends as soon as all known relevant records are identified (i.e., by default --stop-if min=TRUE). During the simulation, two progress indicators track overall progress; the first indicator shows how many relevant records have been found so far, while the bottom indicator counts the total number of records labeled. You can adjust the default settings via additional command-line arguments. A complete overview of options is available by running

asreview simulate --help.

After the simulation finishes, the file MY_SIMULATION.asreview can be used to render performance metrics and obtain (recall) plots via the Insights extension or in the front-end ASReview LAB after importing the project.

### Simulate via the web app

To run a simulation through the ASReview web app, simply create a simulation project. You will be prompted to upload a fully labeled dataset and select a learner—much like setting up a standard screening project. However, in simulation mode, ASReview automatically uses the labels in the dataset to mimic real-time screening interactions. The interface tracks metrics such as recall as records are “screened” and provides visualization, providing immediate insights into the model’s effectiveness. This workflow allows you to experiment with different learners or strategies before committing to a live screening project.

## Extensions

### Insights

The ASReview Insights extension offers tools to plot and compute statistics from one or more ASReview project files. By calculating various performance metrics, Insights enables researchers to compare models, identify outliers, and refine their active learning strategies.

A recall plot visualizes these metrics, often comparing the model’s performance curve to a random baseline and an optimal scenario (where the relevant records appear first). Many of the metrics used in ASReview derive from the literature on active learning and systematic reviews^54^). Within Insights, users can measure the following.

(1) Recall: the proportion of relevant records identified at a given point in the screening process.
(2) Confusion matrix: highlights true positives, false positives, true negatives, and false negatives.
(3) Work saved over sampling (WSS)^53^: quantifies how much effort is saved by screening fewer irrelevant records than a random approach would require.
(4) Extra relevant records found (ERF): assesses the proportion of relevant records found after adjusting for random screening baselines.
(5) Time to discover (TD): represents the fraction of records that must be screened to locate a specific relevant record. This helps identify “hard-to-find” studies buried deep in the ranking.^27^
(6) Average TD (ATD): calculates the mean fraction of records that need to be screened to find all relevant records in the dataset. Because ATD tracks performance across the entire screening process, it eliminates the need for an arbitrary cutoff value. This makes it especially useful for comparing different models.^28^

To use the basic options of the ASReview Insights extension, run

asreview plot recall YOUR_ASREVIEW_FILE.asreview,

where recall is the type of the plot. To obtain the metrics, use

asreview metrics YOUR_ASREVIEW_FILE.asreview.

Metrics can be saved to a file in the JSON format using the flag -o. More options are described in the sections below. All options can be obtained via

asreview plot --help,

asreview metrics --help.

### Makita

The Makita extension (make it automatic) is a workflow generator designed to automate simulation studies in ASReview by leveraging the CLI.^55^^,^^56^ By generating a standardized project structure, pre-written scripts, and documentation, Makita greatly simplifies large-scale simulation experiments—particularly when testing multiple datasets, models, or sets of prior knowledge.

Once Makita and its dependencies, like ASReview and Insights, are initialized, it automatically creates a folder hierarchy for storing data, scripts, and results, along with a customizable README file (e.g., for GitHub). The extension generates all relevant command-line instructions, including random seeds for reproducibility, and provides batch scripts to run the simulations in one go. Researchers can thus efficiently configure and launch a variety of simulations without manually crafting new command lines for each case.

You can create the framework and code for your own simulation study by adding one or more datasets to a data folder and run a template via the CLI:

asreview makita template NAME_OF_TEMPLATE.

Makita offers multiple templates to accommodate different research designs and complexity levels. While the exact list evolves, commonly used templates include the following:

(1) Basic: creates the smallest possible structure for a quick start with one dataset and one simulation scenario. It sets up the directory and scripts for running the same simulation parameters across multiple labeled datasets. When adding more datasets in the *data* folder it sets up the directory and scripts for running the same simulation parameters across multiple labeled datasets.
(2) ARFI: the ARFI template (all relevant, fixed irrelevant) prepares a script for running a simulation study in such a way that for every relevant record one run will be executed with 10 randomly chosen irrelevant records, which are kept constant over runs. When multiple datasets are available the template orders the tasks in the job file per dataset.
(3) Multimodel: the multiple model template prepares a script for running a simulation study comparing multiple models for one dataset and a fixed set of priors (one relevant and one irrelevant record, identical across models).
(4) Prior: the prior template evaluates how a set of custom prior knowledge might affect simulation performance. It processes two types of data in the data folder: labeled datasets to be simulated and labeled datasets to be used as prior knowledge. The filenames of the datasets containing the custom prior knowledge should use the naming prefix prior_[dataset_name]. The template runs two simulations: the first simulation uses all records from the prior_ dataset(s) as prior knowledge, and the second uses a 1 + 1 randomly chosen set of prior knowledge from the non-prior knowledge dataset as a minimal training set. Both runs simulate performance on the combined non-prior dataset(s).

It is also possible to use custom templates. Moreover, Makita also includes scripts to aggregate the simulation outputs into tables and figures, enabling quick comparative analyses. Because of these capabilities, it is particularly suited to high-throughput experimentation or reproducible benchmarking. Up-to-date details and usage guidance are found in the Makita GitHub repository.

### Dory

The ASReview Dory extension expands the range of models available in ASReview. By default, the ASReview core provides several models derived from scikit-learn (https://scikit-learn.org/), including feature extractors such as *onehot* and *tfidf* and classifiers such as *SVM*, *RandomForest*, *NaiveBayes*, and *Logistic*. While ASReview is designed to be accessible to a broad user base, there is also a need to support more advanced and computationally demanding models. To achieve this, deep learning models with more complex dependencies were separated from ASReview core. As a result, the core package depends only on scikit-learn, while Dory relies on larger machine learning libraries such as PyTorch, Keras, and Hugging Face.

With Dory, we introduce two additional pre-sets: ELAS-heavy (MXBAI + SVM), which currently offers the best performance among transformer-based models, and ELAS-lang (E5 + SVM), which provides strong multilingual support with performance comparable to that of other ASReview pre-sets. In addition to these pre-sets, users are able to build custom models using any compatible combination of feature extractor and classifier available through ASReview core and Dory. Once Dory is installed, users can access the following additional models through the graphical user interface (GUI), the CLI, and the API:

(1) Feature extractors: GTR T5, LaBSE, MPNet, Multilingual E5, and MXBAI.
(2) Classifiers: AdaBoost, Neural Network – 2-layer, Neural Network – Dynamic, Neural Network – Warm Start, and XGBoost.

### Community extensions

The ASReview platform is designed to be extensible, allowing developers and researchers to integrate custom functionality via the Python API. Community extensions generally fit into one of the three following categories:

(1) Model extensions: by extending one of ASReview’s base classes—such as asreview.models.classifiers.base for new classifiers or asreview.models.query.base for novel query strategies—users can incorporate new algorithms directly into the screening workflow. The easiest way to do this is to clone the official template and add the new model or feature extraction technique in a dedicated Python file.
(2) Sub-command extensions: these create new entry points for ASReview’s CLI. Each sub-command is a self-contained Python package that can run tasks similar to asreview plot or asreview simulate. To develop a sub-command extension, define a class inheriting from asreview.entry_points.base.BaseEntryPoint and implement the required execute method, which will appear as a new CLI command.
(3) Dataset extensions: this approach integrates additional, potentially domain-specific datasets into ASReview. Once installed, these datasets become accessible directly through the CLI and ASReview LAB’s interface. Under the hood, each dataset extension leverages the entry-point mechanisms of setuptools, allowing the platform to detect and register any newly added resources.

In all three cases, extensions are Python packages that can be installed (e.g., via pip). Once installed, ASReview automatically detects them, allowing both core and community-developed functionalities to coexist and complement one another. This modular design ensures that researchers can rapidly prototype, evaluate, and share new ideas, helping to advance the field of systematic review automation.

## Limitations and future directions

In the following paragraphs, we outline some limitations and detail the steps needed to resolve them, from refining switching heuristics to supporting larger datasets and more sophisticated stopping criteria. Ultimately, these efforts will enable ASReview to realize its full potential as a flexible, human-centric framework for managing the torrent of text data.

Version 2 introduces support for multiple agents in a single project. This flexibility of multiple agents allows users to hand off from one agent to another, for example, moving from a lightweight model to a more advanced context-aware model partway through the review. Although the system architecture already supports automatic switching based on user-defined heuristics, we plan to extend the automatic hand-off functionality in future releases, potentially integrating it with performance metrics so that the system can seamlessly transition between agents without requiring user intervention.

Another future direction involves extending ASReview’s multiagent functionality so that multiple AI agents can operate simultaneously, akin to an ensemble classifier. This “committee” of models would each propose records for screening, leveraging complementary strengths to achieve broader coverage or more robust predictions. However, training multiple models in parallel can become computationally intensive, and it calls for more sophisticated orchestration to handle conflicting or redundant suggestions. Query-by-committee (QBC) is a relevant approach in which each classifier independently ranks unlabeled instances, and the system selects the document on which committee members disagree the most. In contrast, query-by-bagging trains multiple classifiers on randomly sampled subsets of the labeled dataset, and each classifier proposes new instances based on its own query strategy. In our proposed multiagent setup, each agent ranks and suggests documents on its own; the final choice of which agent’s recommendation to present can be made in a round-robin or random manner. Meanwhile, advanced stopping estimators—as discussed by Bron,^43^ building on Chao^57^ and Rivest and Baillargeon^58^—could determine when screening no longer yields meaningful benefits. By integrating a parallel “committee” of agents with data-driven stopping heuristics, future releases of ASReview aim to deliver greater efficiency and accuracy in systematic screening workflows. By integrating a committee of agents with data-driven stopping estimators, future versions of ASReview could provide even more accurate and efficient screening workflows.

Another key improvement in version 2 lies in its hyperparameter optimization. In version 1, tuning was done on just four labeled datasets, and it focused on individual model components in isolation. By contrast, version 2 employs the SYNERGY dataset of 24 labeled systematic reviews, testing combinations of feature extraction, classifier, balancing, and query strategies to find optimal setups for a broader range of topics. This larger-scale approach has significantly boosted performance. Moving forward, the SYNERGY repository will continue to expand with additional labeled sets, allowing further specialization of hyperparameters by research domain and enabling predictive algorithms to suggest configurations likely to perform best on new datasets based on their core characteristics.

ASReview version 1 already offers highly versatile simulation options that enable users to emulate an entire screening process with various model components or initial training sets. In contrast to version 1, simulations in version 2 can include multiple agents in sequence—for example, exploring different hand-off conditions as discussed in our example or validating the set of agents such as those in the SAFE procedure. Despite this flexibility, ASReview version 2 currently assumes that all labels in the fully labeled dataset are accurate reflections of their “true” relevance. A future version could relax this assumption to study the impact of noisy labels, following strategies suggested by Harmsen et al.^29^ This would allow researchers to probe how label inconsistencies affect model performance and to design more robust active learning approaches for systematic reviews.

In version 1, it was already possible to evaluate performance on diverse datasets—ranging from scientific articles to policy reports, news articles, or even business documents from chambers of commerce. Version 2 builds on this versatility by permitting simulations on any feature set, which could include image data from sources like MNIST or Fashion-MNIST. These more creative simulations are currently supported via the CLI and Python API. In a future release, we aim to extend this capability to the web app, so that users can visually inspect and label non-text data directly in their browser.

### Conclusion

The ever-growing influx of textual data—across research publications, policy documents, news articles, and beyond—necessitates systematic screening solutions that can rapidly surface relevant information without compromising on human expertise. While LLMs offer powerful text-processing capabilities, relying on them alone raises concerns about factual accuracy and domain-specific reliability. ASReview version 2 embraces AI as a “super assistant,” amplifying human decision-making rather than replacing it. By designing a system that keeps the reviewer firmly in control, we aim to accommodate a wide spectrum of applications while maintaining rigorous quality standards. With its new multiagent architecture and flexible simulation modes, it lays the groundwork for the next major leap in systematic screening. Ultimately, we see these enhancements paving the way for advanced features—such as dynamic stopping criteria, real-time collaboration, and even more robust support for varied data types—where human expertise remains central.

## Resource availability

### Lead contact

Requests for further information and resources and reagents should be directed to and will be fulfilled by the lead contact, Rens van de Schoot (a.g.j.vandeschoot@uu.nl).

### Materials availability

This section is not applicable to this work.

### Data and code availability

All original code of the software has been deposited at Zenodo (ASReview LAB,^59^ DORY,^60^ MAKITA,^56^ and INSIGHTS^61^) and is publicly available as of the date of publication. Simulation study scripts and output are available at OSF.^47^

## Acknowledgments

The 10.13039/501100003246Dutch Research Council funded this project under grants no. 406.22.GO.048 and VI.C.231.102. Part of the work was executed within the scope of the EU project “IMPROVE,” supported by the Innovative Health Initiative Joint Undertaking (IHI JU) under grant agreement no. 101132847.

## Author contributions

Conceptualization, J.d.B., P.L., J.T., and R.v.d.S.; methodology, J.d.B., P.L., J.T., and T.v.d.K.; investigation, J.d.B., P.L., J.T., T.v.d.K., A.D., and B.Y.; writing – original draft, R.v.d.S.; writing – review & editing, J.d.B.; funding acquisition, R.v.d.S.; resources, J.d.B.; supervision, J.d.B. and R.v.d.S.

## Declaration of interests

The authors declare no competing interests.

## Declaration of generative AI and AI-assisted technologies in the writing process

During the preparation of this work, the authors used Grammarly and ChatGPT in order to improve grammar and sentence structures. After using this tool or service, the authors reviewed and edited the content as needed and take full responsibility for the content of the publication.
